# Semantic validation of the short versions of the Empathy-Systemizing Quotient Scales

**DOI:** 10.1590/1518-8345.2606.3044

**Published:** 2018-10-11

**Authors:** Mirella Castelhano-Souza, Isabel Amélia Costa Mendes, José Carlos Amado Martins, Maria Auxiliadora Trevizan, Valtuir Duarte Souza-Júnior, Simone de Godoy

**Affiliations:** 1 Universidade de São Paulo, Escola de Enfermagem de Ribeirão Preto, PAHO/WHO Collaborating Centre for Nursing Research Development, Ribeirão Preto, SP, Brazil.; 2 Escola Superior de Enfermagem de Coimbra, Coimbra, Portugal.; 3 Fundação Hemominas, Hemocentro Regional de Uberaba, Uberaba, MG, Brazil.

**Keywords:** Nursing, Empathy, Students Nursing, Surveys and Questionnaires, Nursing Staff, Behavior

## Abstract

**Objective:**

to perform the semantic validation of the short versions of the *Empathy-Systemizing Quotient Scales*, intended to measure the empathetic and systemizing profiles of individuals. The scales originated in Cambridge and were validated in Portugal, and were assessed for their psychometric properties.

**Method:**

methodological study included the scales’ semantic validation (content validity) and verification of their psychometric properties (internal consistency). Five judges participated in the semantic validation. The Content Validity Index was calculated, a pretest was conducted with 18 undergraduate nursing students, and, finally, the scales were applied to a sample.

**Results:**

the sample was composed of 215 undergraduate nursing students, 186 (86.51%) of whom were women aged 21 years old, on average. The scales presented good internal consistency with global Cronbach’s alphas equal to 0.83 and 0.79 for the *Empathy Quotient* and the *Systemizing Quotient,* respectively. Correlations between the scales and subscales of the *Empathy Quotient* and *Systemizing Quotient* were all positive and significant according to the Pearson correlation coefficient.

**Conclusion:**

the scales are reliable and valid to measure the empathetic and systemizing profile of undergraduate nursing students and the final version was named *“*versões curtas das *Escalas de Medição do Quociente de Empatia/Sistematização - Brasil*” [short versions of the *Empathy-Systemizing Quotient Scales* - Brazil].

## Introduction

Empathy, the ability to identify the thoughts and emotions of people, is an essential component of social interactions, enabling one to perceive the feelings of people, infer their intentions and understand their behaviors^1^. It is, however, important to note that empathy may vary according to individuals’ personalities and emotional states. A person who is emotionally shaken will have more difficulty understanding the point of view of others^2^ ^-^ ^3^. Empathy is essential to facilitating effective social interactions and is more developed among women^2^ ^-^ ^4^.

Systemizing is a cognitive ability that enables understanding the variables of a system and its rules so that individuals are able to predict and control a system’s behavior^1^. It is the ability to grasp information and manipulate it in different manners. When an individual follows the rules, his/her brain focuses on observing the details and functioning of systems. These observers tend to be methodical, with such a profile being more predominant among men^5^.

The empathy-systemizing theory (E-S) was developed to distinguish between these two opposite concepts and test them with different people to identify their profiles through social behavior. Even though these are opposed concepts, they are similar in the sense that they give meaning to events and allow reliable predictions. They can be seen as two cognitive dimensions that determine a female or a male brain. Everyone has empathic and systematic skills; however, some people tend to develop one of the two in a greater extent and others even achieve a balance between the two^6^ ^-^ ^7^.

There are five types of brains, namely: Type E, in which empathy is more developed than systemizing and is more commonly a “female brain”; Type S, in which systemizing is more developed than empathy and is more commonly a “male brain”; Type B, in which systemizing and empathy are balanced, the so-called “balanced brains”; the Extreme E, in which empathy is much more developed than systemizing, called “mind-reading”; and the Extreme S, in which systemizing is much more developed than empathy, called “mind-blindness”^6^ ^,^ ^8^. Note that not all women have a “female brain” and not all men have a “male brain”; this classification is used only because it represents the majority of people^3^ ^,^ ^9^.

From an empathetic perspective, the focus is on the person’s mental state that includes this emotion. If an individual presents a very low level of emotion, it may be she has some mental disorder, such as autism, and it is a simple way to explain social and communicative obstacles, while a high level of systemizing is expressed through repetitive behaviors and resistance against the new. Therefore, while empathetic individuals are emotionally concerned with others, systematic people are concerned with their emotional control and their own interests^3^ ^,^ ^10^.

In 2003, researchers from the Department of Experimental Psychology and Psychiatry at the University of Cambridge, United Kingdom, developed the *scales Empathy Quotient (EQ) and Systemizing Quotient (SQ)*. These scales were initially tested among individuals with autism, Asperger’s syndrome or high-functioning autism, but presented normal intelligence quotient (IQ). Individuals with these disorders present low social interaction and face problems with communication processes. The scales presented the expected results in this population^2^ ^,^ ^5^ and thereby have been applied in diverse types of population around the world.

Initially, the two scales were applied separately, each with 60 multiple-choice questions: the empathy scale contained 40 questions addressing empathy and 20 complementary questions that were only intended to distract the responder. The systemizing scale was constructed using the same rationale and number of questions^2^ ^,^ ^5^.

After four years, short versions of these scales were created: the *Empathy Quotient (EQ-Short)*, containing 22 questions, and the *Systemizing Quotient (SQ - Short)* with 25 questions. They were examined using psychometric analyses and the internal consistency was greater than that found for the original versions, so that both versions are reliable and appropriate to measure individual differences concerning empathy and systemizing^11^.

In Brazil, there are several scales that measure empathy profiles but none measure the systemizing profile. Considering the important role empathy plays in the interpersonal relationships among healthcare workers and between them and patients and the community, we decided to validate the short versions of the *Empathy Quotient (EQ)* and *Systemizing Quotient (SQ) scales*. When we first contacted the primary author of the original version in English^2^, he informed us that the questionnaires had already been validated in many languages, including Portuguese as spoken in Portugal. For this reason, we decided to conduct a semantic adaptation of the version that had been already validated in Portugal for Brazilian Portuguese.

In 2011, the short versions were validated for Portuguese as spoken in Portugal and were called *Escalas de Medição do Quociente de Empatia/Sistematização* [*Empathy/Systemizing Quotient Scales*]. Exploratory factor analysis was performed for both versions and reasonable results, similar to those obtained for the original versions, were found: Cronbach’s alpha equal to 0.90 was found for the *EQ* and equal to 0.89 for the *SQ*. Four domains were identified in the Portuguese version of the Empathy Quotient, namely: “Cognitive Empathy (CE)”, “Emotional Reactivity (ER)”, “Social Skills (SS)” and “Empathy Difficulties (ED)”. Two domains were found in the Systemizing Quotient version*,* namely: “Processes (P)” and “Content (C)”. The *EQ* initially had 22 questions; however, one item of the SS domain was removed because it scored poorly in the analysis of the Cronbach’s alpha coefficient and removing it did not change the global Cronbach’s alpha^1^ ^,^ ^12^.

These scales have been successfully used in various countries to measure both empathy and systemizing profiles. 

Thus, this study’s objective was to perform semantic validation and assess the psychometric properties of the short versions of the *Escalas de Medição do Quociente de Empatia/Sistematização* [Empathy/Systemizing Quotient Scales].

## Method

This methodological study includes the semantic validation of the short versions of the *Escalas de Medição do Quociente de Empatia/Sistematização* [Measurement *Scales of Empathy/Systemizing Quotient*] for Brazilian Portuguese. It was divided into two phases: 1) semantic validation (content validity) and 2) assessment of psychometric properties (internal consistency).

First, we contacted the researcher who validated the scales’ short versions in Portugal^6^, who sent us the questionnaire and authorized the semantic validation in Brazil.

In this phase, we conducted Face and Content Validity to assess semantic, experiential, idiomatic and conceptual equivalences. Five judges collaborated in the study: three Brazilian nurses, one Portuguese nurse, one internationalist, and one lawyer. All of these had teaching experience and were fluent in both languages. They classified the items in the questionnaire as being appropriate or not appropriate, and the Content Validity Index (CVI) was calculated. The items with CVI equal to 100% were definitively kept in the questionnaire. The items with a CVI index lower than 80% were changed and reassessed by the judges, who agreed with all the changes implemented^13^. 

The scales were then pretested with 18 undergraduate nursing students during a meeting, simulating the expected conditions of future application. The students received clarification regarding the purpose of the pretest and after they finished completing the questionnaires, they were encouraged to verbalize their doubts. No changes were suggested, so the scales were considered to be understandable for the target-public. Hence, the final short versions of the scales were named: *“Escalas de Medição do Quociente de Empatia/Sistematização -* Brasil*”* [*Empathy-Systemizing Quotient Scales* - Brazil].

Based on the model used by the authors of the short versions validated in Portugal^6^ ^,^ ^12^, in the second phase, the scales’ psychometric properties were assessed using analysis of reliability, by measuring the items’ internal consistency, calculated with Cronbach’s alpha. This coefficient is recommended because it reflects the degree of covariance among items; values greater than 0.70 are acceptable, as they reflect a high degree of internal consistency^14^. Pearson’s correlation coefficient was used only to verify the relationship between the scales’ total scores and among the factors of scales *EQ* and *SQ*.

The level of significance was established at 0.05. We assumed that correlations would be positive and significant between the domains (Cognitive Empathy, Emotional Reactivity, Social Skills, and Empathetic Difficulties) and between the domains and the *Empathy scale’s* total score and between the factors (Content and Processes) and the *Systemizing scale’s* total score. Pearson’s correlation was performed according to the following classification: *r*<0.2 means very low association; low association when between 0.2 and 0.39; moderate when between 0.4 and 0.69; high when between 0.7 and 0.89; and very high when between 0.9 and 1.0^15^.

The study addressed a sample of 215 undergraduate nursing students from a public university located in the interior of São Paulo, Brazil. Data were collected from October to November 2016 with students attending from the 1^st^ to the 5^th^ years, enrolled in the bachelor’s degree and teaching diploma degree programs. Data were collected on days all students were taking classes in the same classroom. The study’s objective was clarified and presented by the researcher who invited the students to participate. Free and informed forms were handed out together with the questionnaires, which the students were asked to return after completing them. In addition to the *Empathy Quotient (EQ)* and *Systemizing Quotient (SQ)* scales, sociodemographic information was also collected to characterize the participants (sex, date of birth, program and year). Approximately 15 minutes were necessary to complete the questionnaires. 

The study was initiated after approval was obtained from the authors of the original questionnaires and the author of the version validated for Portuguese from Portugal, as well as from the Institutional Review Board (Opinion report 191/2016). 

The *EQ* has 21 items distributed into four domains: Cognitive Empathy (9,12,18,19,20); Social Skills (1,6,10,13,15); Emotional Reactivity (2,8,14,17,21) and Empathetic Difficulties (3,4,5,7,11,16). The *SQ* presents 25 questions distributed in the factors “Content” (3,4,7,8,9,10,11,12,15,17,19,20) and “Processes” (1,2,5,6,13,14,16,18,21,22,23,24,25). The reversed items were: *EQ* (3,4,5,7,11,16), and *SQ* (3,4,7,9,10,11,12,15,17,19,20,23,25).

Answers to the items are listed on a four-point Likert scale, where (1) refers to “Totally Agree”; (2) means “Partially Agree”; (3) “Partially Disagree”; and (4) “Totally Disagree”. The participant can score (0) zero (if answers are not aligned with empathy/systemizing); score (1) (if answers are partially aligned with empathy/systemizing); or (2) (if answers are totally aligned with empathy/systemizing). That is, each item is scored (2,1,0,0), such that total score can range from 0 to 42 points in the *EQ* and from 0 to 50 points in the *SQ*.

Data were double entered into Excel spreadsheets to verify consistency and then transferred to the IBM SPSS, version 24 (2016), in which descriptive analyses were performed to characterize the students and the scores obtained on the *EQ* and *SQ* scales. 

## Results

Of the 215 participants, 186 (86.5%) were women aged from 17 to 48 years old; 21 years old on average (SD= 3.21).

The scales presented global Cronbach’s alphas equal to 0.83, for the *Empathy Quotient*, and 0.79 for the *Systemizing Quotient* (Table 1).

**Table 1:** Total reliability and reliability per domain of the short versions of the scales *Measuring Scales of Empathy-Systemizing Quotien* *t* - Brazil according to the Cronbach’s alphas, Ribeirão Preto - SP, Brazil, 2016

Factors	Items	Cronbach’s alpha	Interval	Median	Mean (Standard Deviation)
EQ*					
Social Skills	1,6,10, 13,15	0.70	2-10	7.0	6.4 (2.1)
Empathetic Difficulties	3,4,5,7,11,16	0.57	0-12	6.0	5.8 (2.5)
Cognitive Empathy	9,12,18,19,20	0.78	0-10	4.0	4.4 (2.5)
Emotional Reactivity	2,8,14,17,21	0.73	0-10	7.0	6.2 (2.4)
Total Scale	21	0.83	5-42	23.0	22.8 (7.0)
SQ^†^					
Content	3,4,7,8,9,10,11,12,15,17,19,20	0.64	2-24	8.0	8.1 (3.8)
Processes	1,2,5,6,13,14,16,18,21,22,23,24,25	0.69	0-24	12.0	12.3 (4.4)
Total Scale	25	0.79	7-47	20.0	20.4 (7.2)

*Empathy Quotient; †Systemizing Quotient

Correlations between the scores of the short versions of the *Empathy-Systemizing Quotient Scales*- Brazil were moderate between “Empathetic Difficulties” and “Emotional Reactivity” (*r*=0.406; *P*=0.000) and between “Cognitive Empathy” and “Emotional Reactivity” (*r*=0.515; *P*=0.000); were between “Social Skills” and “Emotional Reactivity” (*r*=0.391; *P*=0.000), between “Empathetic Difficulties” and “Cognitive Empathy” (*r*=0.358; *P*=0.000) and between “Social Skills” and “Emotional Reactivity” (*r*=0.391; *P*=0.000); and very low between “Social Skills” and “Empathetic Difficulties” (*r*=0.141; *P*=0.039) (Table 2).

**Table 2:** Pearson correlation coefficient between the domains of the short versions of the *Empathy-Systemizing Quotient Scales* - Brazil, Ribeirão Preto - SP, Brazil 2016.

	ED*		CE^†^		ER^‡^		EQ^§^		P^||^		SQ^¶^	
	*r*	P	*r*	P	*r*	P	*r*	P	*r*	P	*r*	P
Social Skills	.141	.039	.583	.000	.391	.000	.684	.000				
Empathetic Difficulties			.358	.000	.406	.000	.668	.000				
Cognitive Empathy					.515	.000	.826	.000				
Emotional Reactivity							.783	.000				
Content									.498	.000	.885	.000
Processes											.845	.000

*ED Empathetic Difficulties; †CE Cognitive Empathy; ‡ER Emotional Reactivity; §*EQ* *Empathy Quotient*; ||P Processes; ¶*SQ* *Systemizing Quotient*

## Discussion

The psychometric tests revealed a high correlation between variables, showing the sample is adequate. The global Cronbach’s alphas of the *EQ/SQ* were compared to those obtained for the short versions of the original questionnaires (*EQ’s* alpha=0.90 and *SQ’s* alpha=0.89) and the short versions validated in Portugal (*EQ’s* alpha=0.85 and *SQ’s* alpha=*0.*72), which demonstrated that the Brazilian versions presented reliability and internal consistency, as a high correlation was found among the variables^1^ ^-^ ^2^ ^,^ ^5^ ^,^ ^11^ ^-^ ^12^.

The Cronbach’s alpha values revealed reasonable internal consistency for the EQ factors, low internal consistency for the *SQ* factor “Contents”, and reasonable internal consistency for the *SQ* factor “Processes”. Comparison between the factors found in the short version validated by the Portuguese researchers and that were validated in Brazil returned similar results^1^ ^,^ ^12^.

The correlations were all positive and significant, confirming our hypothesis. Comparing the correlation results with the findings of the Portuguese researchers^6^, similar results were found: “CE” and “SS” *r*= .606; “CE” and “ER” *r*= .559; “SS” and “ER” *r*= .538; “ED” and “SS” *r*= .302. In regard to the correlation per factors related to the *EQ*, good results were found for “ER” (*r*= .783; *P*=0.000), “SS” (*r*= .826; *P*=0.000) and moderate results were found between “ED” (*r*= .668; *P*=0.000) and “SS” (*r*= .684; *P*=0.000). The Pearson’s correlation results concerning the *Systemizing Quotient* were moderate between “Processes” and “Content” (*r*= ,498; *P*=0.000), and when intercalating factors with the *SQ*, good results were also found, such as “P” (*r*= .845; *P*=0.000) and “C” (*r*= .885; *P*=0.000).

Various studies tested the psychometric properties of the scales using diverse populations, such as undergraduate students in the fields of exact and human sciences^12^ ^,^ ^17^ ^-^ ^18^, undergraduate students of different programs^18^, nursing undergraduate students^19^ ^-^ ^20^, individuals with depersonalization disorders^21^, with autism^22^ ^-^ ^26^, children and adults with attention deficit and hyperactivity disorder^27^, and all found good reliability. The short versions of the *Empathy-Systemizing Quotient Scales* have been applied to various different populations and this study confirms the validity and reliability of these versions among undergraduate nursing students. 

The semantic validation of this instrument is a valuable contribution to the Brazilian literature in the field of empathy and its application in distinct populations, such as in the field of nursing, and in other health professions in which there is human contact, is essential to care delivery and a very useful resource to be explored in the management of people and in teaching. Thus, the validated scales can support various studies and contribute to the clinical, managerial, and pedagogical practices of nurses. 

## Conclusion

The short versions of the *Empathy-Systemizing Quotient Scales* - Brazil are valid and reliable to measure the empathetic and systemizing profile of undergraduate nursing students. These scales can be applied separately because they are independent. A limitation of this study is that it was conducted in a single institution and with a single population. Future studies should test the scales’ psychometric properties in other Brazilian populations.
